# How well do participants in clinical trials represent the U.S. population with chronic neck or back pain?

**DOI:** 10.1186/s12891-024-07524-9

**Published:** 2024-05-27

**Authors:** Brent D. Leininger, Pamela Jo Johnson, Gert Bronfort, Karen M. Kuntz, Eva Enns, James S. Hodges, Roni Evans

**Affiliations:** 1 Integrative Health and Wellbeing Research Program Earl E. Bakken Center for Spirituality & Healing, University of Minnesota, Mayo Memorial Building C504, 420 Delaware Street, Minneapolis, MN 55414 USA; 2 Department of Public Health, North Dakota State University, 640R Aldevron Tower, 1455 14th Ave N, Fargo, ND 58102 USA; 3 Division of Health Policy & Management School of Public Health, University of Minnesota, 420 Delaware St SE, MMC 729 Mayo, Minneapolis, MN 55455 USA; 4 Division of Biostatistics, School of Public Health, University of Minnesota, 2221 University Ave SE, Room 200 University Office Plaza, Minneapolis, MN 55414 USA

**Keywords:** Chronic pain, Clinical trials, Health disparities, Generalizability

## Abstract

**Background:**

Randomized clinical trials (RCTs) are the gold standard for assessing treatment effectiveness; however, they have been criticized for generalizability issues such as how well trial participants represent those who receive the treatments in clinical practice. We assessed the representativeness of participants from eight RCTs for chronic spine pain in the U.S., which were used for an individual participant data meta-analysis on the cost-effectiveness of spinal manipulation for spine pain. In these clinical trials, spinal manipulation was performed by chiropractors.

**Methods:**

We conducted a retrospective secondary analysis of RCT data to compare trial participants’ socio-demographic characteristics, clinical features, and health outcomes to a representative sample of (a) U.S. adults with chronic spine pain and (b) U.S. adults with chronic spine pain receiving chiropractic care, using secondary data from the National Health Interview Survey (NHIS) and Medical Expenditure Panel Survey (MEPS). We assessed differences between trial and U.S. spine populations using independent t-tests for means and z-tests for proportions, accounting for the complex multi-stage survey design of the NHIS and MEPS.

**Results:**

We found the clinical trials had an under-representation of individuals from health disparity populations with lower percentages of racial and ethnic minority groups (Black/African American 7% lower, Hispanic 8% lower), less educated (No high school degree 19% lower, high school degree 11% lower), and unemployed adults (25% lower) with worse health outcomes (physical health scores 2.5 lower and mental health scores 5.3 lower using the SF-12/36) relative to the U.S. population with spine pain. While the odds of chiropractic use in the U.S. are lower for individuals from health disparity populations, the trials also under-represented these populations relative to U.S. adults with chronic spine pain who visit a chiropractor.

**Conclusions:**

Health disparity populations are not well represented in spine pain clinical trials. Embracing key community-based approaches, which have shown promise for increasing participation of underserved communities, is needed.

**Supplementary Information:**

The online version contains supplementary material available at 10.1186/s12891-024-07524-9.

## Background

Randomized clinical trials (RCTs) are recognized as the gold standard study design for assessing treatment effectiveness. However, generalizability issues are common among RCTs and include the ability to recruit a representative population, reliance on protocolized interventions, limitations in the number of treatments assessed, and a limited time horizon for assessing effects [1]. A key component of generalizability is the trial patient population and how well they represent the population that will receive the treatment in clinical practice [2]. The final trial population is shaped by the sampling or recruitment plan (e.g., recruit from secondary clinics or general population), inclusion and exclusion criteria (e.g., exclusion of patients with comorbidities), individuals’ compliance with trial procedures prior to randomization, their preferences for treatment (must be willing to accept all treatments under study), and their willingness to comply with the extensive data collection that is common with RCTs. These limitations can theoretically be minimized through pragmatic RCTs which recruit participants from the general population, use broad inclusion and limited exclusion criteria, use common treatment alternatives, and mimic treatment delivery in “real world” healthcare settings [3].

The representativeness of RCT populations is an important issue that potentially limits the ability of RCT findings to influence clinical practice and policy. If RCT populations differ in important ways from the general population, researchers can use this information to design better sampling and recruitment strategies to reach under-represented populations. Also, the relationship between factors on which the populations differ and study outcomes can be explored to better estimate the potential impact on external validity. Differences in the RCT and general population only impact the external validity of a study if the treatment effects found in the RCT are modified by the variable of interest. For instance, if the populations differ in terms of household income, but treatment effects are not influenced by household income, then the external validity of the RCT’s findings are not compromised [4]. On the other hand, if RCT populations are shown to be representative of the general population, arguments against their external validity can be better addressed, and their findings may be more easily adopted into clinical practice and public policy. Currently, there are no analyses assessing the representativeness of populations enrolled in RCTs for spinal pain in the United States (U.S.) with respect to the broader U.S. spine pain population. The clinical course for back pain intensity has been shown to be similar in RCTs and observational studies; however, analyses comparing demographic and clinical characteristics of the general U.S. spine pain population to individuals participating in clinical trials or observational studies are lacking [5].

This project’s purpose is to evaluate the generalizability of chronic spinal pain RCT populations that were used for an individual participant data meta-analysis of the cost-effectiveness of spinal manipulation for spine pain. Generalizability was assessed by comparing demographic and clinical characteristics to the U.S. spine pain population using data from national health and healthcare surveys. In addition, since spinal manipulation was performed by chiropractors in the clinical trials, the study aimed to assess for differences in demographic and clinical characteristics in the U.S. spine pain population based on chiropractic use.

## Methods

We conducted a retrospective secondary analysis of RCT data, comparing trial participants’ socio-demographic characteristics, clinical features and healthcare utilization to a representative sample of (a) U.S. adults with chronic spine pain and (b) U.S. adults with chronic spine pain receiving chiropractic care, using secondary data from the National Health Interview Survey (NHIS) and Medical Expenditure Panel Survey (MEPS). We also assessed demographic and clinical predictors of chiropractic use in U.S. adults with chronic spinal pain using NHIS and MEPS data.

### Populations

We compared RCT enrollees with chronic spinal pain, 1,444 participants in eight clinical trials, included in an individual participant data meta-analysis project assessing the cost-effectiveness of spinal manipulation for back or neck pain [6], to a representative sample of the US population with chronic spinal pain from the NHIS and MEPS. Previous research has found differences in demographic and clinical characteristics in adults with low back and neck pain receiving complementary and integrative care, including chiropractic care [7, 8]. Since all of the RCTs included chiropractic spinal manipulation, we also completed analyses limiting the NHIS and MEPS samples to individuals receiving care from a chiropractor in the past year. We used a sample of eight clinical trials that collected similar sociodemographic and clinical characteristics and measured them consistently.

#### RCT inclusion/exclusion criteria, setting, and recruitment methods

We included participants from eight clinical trials conducted between 1994 and 2012 [9–16]. We included adults 18 or older with weekly, persistent, mechanical, non-specific neck or low back pain, with or without radiating extremity symptoms, lasting 12 weeks or longer. All trials required self-reported pain severity to be ≥ 3/10. Individual trial inclusion criteria for age were 12–18 [13], 18–65 [11, 12], 20–65 [9], 21 or older [10], or 65 and older [14, 15]. Other standard inclusion criteria included having a stable medication plan and no ongoing spinal treatment at the time of enrollment. Common exclusion criteria included pregnancy, current or pending litigation, the inability to read or comprehend English, substance abuse, history of surgical spinal fusion, progressive neurological deficits, inflammatory spinal arthropathies, spinal fractures, metastatic disease, blood clotting disorders, and severe disabling health problems (e.g., organ failure). All of the clinical trials were performed in a university-affiliated research clinic in the Minneapolis, Minnesota metropolitan region. Five of the trials were performed exclusively in Minnesota and two were multi-center studies with additional sites in Portland, Oregon [13] or Davenport, Iowa [10]. All trials recruited participants from the general population primarily through mass mailings. Other recruitment strategies included advertisement in newspapers, social media, television, radio, and community posters.

#### NHIS & MEPS

We used data from the NHIS accessed through the IPUMS NHIS database [17]. The NHIS is an annual, cross-sectional, in-person household survey used to monitor the health of US citizens [18]. Approximately 35,000 to 40,000 households including 75,000 to 100,000 individuals are interviewed annually with a response rate of 80–90%. NHIS uses a complex sampling design with oversampling of Black, Hispanic, and Asian populations to ensure representativeness of the US civilian noninstitutionalized population. We pooled data from the 2001–2010 NHIS resulting in a total of 883,541 unweighted adult observations. Most trial activities were conducted between 2001 and 2010 which is why these years were used for defining the U.S. sample. The NHIS is also the sampling frame for the MEPS. MEPS collects data on health services utilization and costs as well as health status and socio-demographic characteristics. Approximately 12,000 to 15,000 households with 32,000 to 37,000 individuals complete the MEPS annually with response rates between 58 and 71%. MEPS uses an overlapping panel design with five rounds of interviews occurring every five to six months over a two-and-a-half-year period to capture longitudinal changes in health and expenditures. We used data from the 2002–2010 MEPS resulting in 309,620 observations. MEPS data from 2001 had a survey design that differed substantially from 2002 to 2010 data and was omitted (e.g., non-compatible primary sampling units and independent variance strata).

### Measures

#### Chronic spine pain and chiropractic use variables

In the IPUMS NHIS database, we identified individuals with functional limitations (e.g., difficulty shopping, participating in social activities, walking, etc.) due to a neck or back problem that lasted at least 3 months using the variable FLBACKC (See Appendix Table 1). NHIS also collects information on types of health care providers seen in the past 12 months. We used the chiropractic use variable (SAWCHIR) to identify individuals with chronic spine pain receiving chiropractic care. For MEPS, we identified individuals with healthcare expenditures for spine problems in at least 2 of the 5 interviews over the 2.5 years the MEPS is administered. We used the Clinical Classification Code 205 within the Medical Conditions File to identify individuals with healthcare visits for spondylosis, intervertebral disc disorders, or other back problems. The number of office visits to a chiropractor (MEPS variable CHIROVISIT) was used to identify individuals with chronic spine problems receiving chiropractic care.

#### Demographic and health characteristics

We selected socio-demographic and clinical characteristics based on the minimal data set recommendations from the National Institute of Health’s task force on research standards for chronic low back pain [19], as well as on availability of comparable measures within the trials and NHIS or MEPS. We included the following socio-demographic variables: age, sex, race (Alaskan Native or American Indian, Asian or Pacific Islander, Black, White, Multiple races, Other race), ethnicity (Hispanic or not Hispanic), employment (yes or no), education (less than high school, high school, some college, Associate or Technical school degree, Bachelor degree, Post-graduate or professional degree), and household income ($0-$35k, $35k-$75k, $75k+). Clinical measures included a body mass index indicator (underweight: <18.5, healthy weight: 18.5–24.9, overweight: 25-29.9, obese: 30 or more), duration of neck or back problem (1 year or less, 1–5 years, 5–10 years, over 10 years), presence of radiating leg or arm pain, diabetes, current smoker, current alcohol use, the SF-12 physical component summary score (PCS, constructed so a normative score for U.S. population is 50 with standard deviation of 10), the SF-12 mental component summary score (MCS, constructed so a normative score for U.S. population is 50 with standard deviation of 10), and quality of life scores. Quality-adjusted life years (QALYs) were derived using the SF6D scoring system for describing health states using SF-12 data from MEPS and SF-36 data from the clinical trials. We calculated QALYs using weights from a study assessing U.S. preferences for SF6D health states with discrete choice experiments [20]. SF-12 and SF6D measures were computed using MEPS data. All other socio-demographic and clinical characteristics were computed using NHIS data. Details on NHIS and MEPS variables used for the analysis are provided in Appendix Table 1.

### Analyses

All analyses used NHIS (*n* = 15,312) and MEPS (*n* = 12,679) data from survey respondents with chronic spine pain and no missing items for our chosen sociodemographic and clinical measures. The analysis consisted of four stages. First, we calculated point estimates (i.e., means, proportions), standard errors and confidence intervals for socio-demographic and clinical characteristics in both clinical trial and national survey participants with chronic spine pain. Sampling weight variables provided by MEPS and NHIS were used to account for the unequal probability of selection and were divided by the number of years used for the analyses as recommended by IPUMS [21]. We used design variables (e.g. strata and primary sampling unit) to account for the stratification and clustering of the complex multi-stage survey design. Standard errors for MEPS and NHIS data were estimated using the Taylor-series linearization method [22]. Second, we assessed differences between trial and U.S. spine populations using independent t-tests for means and z-tests for proportions. Third, we conducted multivariable logistic regression to assess differences in socio-demographic and clinical characteristics based on chiropractic use in the U.S. population with chronic spine pain using NHIS data. The outcome was chiropractic use and predictors were socio-demographic and clinical characteristics. Characteristics not included in all ten years of NHIS data were excluded from this analysis (i.e., history of arthritis or depression). Association of chiropractic use with SF-12 and QALYs were assessed using multivariate logistic regression adjusted for age, sex, race, and ethnicity from MEPS. Finally, we compared trial participants to the U.S. population with chronic spine pain that reported chiropractic use using independent t-tests for means and z-tests for proportions. Statistical significance for all analyses was determined using two-sided tests with a threshold of *p* ≤ 0.05. The magnitude of differences was depicted using effect sizes. We used Cohen’s h to calculate effect sizes for differences in proportions $\left(\right|2*\left(arcsin\sqrt{{P}_{1}}\right)-2*\left(arcsin\sqrt{{P}_{2}}\right)\left|\right)$ and standardized mean differences to calculate effect sizes for differences in means$\left(\left|\frac{mean \; difference}{standard \; deviation \; in \; NHIS \; or \; MEPS \; sample}\right|\right)$ [23]. We used Cohen’s suggested definitions for interpretating effect size changes (Small = 0.20; Medium = 0.50; Large = 0.80) [23]. As two of the trials only enrolled adults 65 or older, we assessed the impact of stratifying analyses comparing trial and U.S. populations by age (18–64 and 65 or older) by comparing findings from stratified and unstratified analyses. We reported variables where the effect size interpretation changed stratified by age. We used Stata 13.1 for all analyses, in particular we used the svy commands to account for the complex sample design of the NHIS and MEPS (i.e., unequal probability of selection, clustering, and stratification).

## Results

Baseline demographic and health characteristic data were available from the vast majority of participants in all 8 RCTs. Socio-demographic and clinical characteristics selected for the project were available in all 8 RCTs except for education, history of headaches, and SF-36 measures in the trial of 12–18 year olds [13] and alcohol use in one of the chronic neck pain trials [9]. Within the RCTs, nearly complete data (97% or higher) was available for all measures except for household income (83%) and alcohol use (87%). National survey data was limited to participants with complete data for all demographic and health characteristics. Stratifying analyses comparing the trial and U.S. populations by age had little impact, with the effect size interpretation changing for only one variable (employment).

### Trial participants relative to the US population

Table 1 presents socio-demographic and clinical characteristics for the U.S. and clinical trial populations. The analytic sample from the NHIS included 15,312 adults with functional limitations due to a persistent neck or back problem, the MEPS sample included 12,679 adults with chronic spine-related healthcare expenditures, and the clinical trials included 1,444 adults with chronic neck or back pain. The NHIS and MEPS samples were representative of approximately 11.5 and 15.6 million U.S. adults, respectively. The difference in samples between NHIS and MEPS is due to differences between the two data sources in how chronic spine pain was defined (functional limitation due to a back or neck problem in NHIS and multiple healthcare visits over 2.5-year period for back or neck condition in MEPS). The clinical trials had a higher percentage of older adults relative to the U.S. population (17% more 65 to 84 year olds) because they included two trials that focused solely on older adults [14, 15]. The percentage of females in the trials was larger than in the U.S. population with chronic spinal pain (3.6% more). For other socio-demographic characteristics, several important differences were observed. The clinical trials had a higher percentage of White participants (8.5% more) and fewer Asian or Pacific Islander (1% less), Black (7.3% less), and Hispanic (8% less) participants. A higher proportion of trial participants were employed (24.9% more), earned a Bachelor’s (17.8% more), post-graduate, or professional degree (9.9% more), and had higher household incomes (14.3% more with $35,000–74,999). For clinical characteristics, a higher percentage of trial participants had a healthy weight (3.9% more), a shorter pain duration (11.2% more with duration less than one year), and no radiating arm or leg pain (13.2% more), diabetes (6.8% more), or smoking (21.6% more). Trial participants reported a higher level of physical (2.5 higher SF-12/36 physical health score) and mental health (5.3 higher SF-12/36 mental health score) than the U.S. population according to the SF-12 as well as higher quality-adjusted life years (0.07 higher). There were no differences in alcohol use. Most differences were small in magnitude. Differences in age, ethnicity, income, and mental health were small to medium in magnitude and differences in education, employment, and smoking status had medium effect sizes.

**Table 1:** U.S. Population with chronic spine pain using NHIS and MEPS relative to trial participants

	U.S. Population		Trial Population			Trial – US Population		
	% or Mean (SD)	SE	% or Mean (SD)	SE	*N*	Difference	*P*-value	Effect Size
**Age†**								
18 to 30	10.6%	0.004	7.0%	0.007	101	-3.6%	< 0.001	0.13
31 to 50	38.8%	0.005	32.8%	0.012	473	-6.0%	< 0.001	0.13
51 to 64	30.0%	0.005	23.5%	0.011	339	-6.6%	< 0.001	0.15
65 to 84	18.5%	0.004	35.9%	0.013	518	17.3%	< 0.001	0.40
85 and above	2.1%	0.001	0.8%	0.002	12	-1.2%	0.0013	0.11
**Sex†**								
Male	44.4%	0.005	40.7%	0.013	588			
Female	55.6%	0.005	59.3%	0.013	856	3.6%	0.009	0.07
**Race†**								
Alaskan Native, or American Indian	1.4%	0.001	0.9%	0.002	13	-0.5%	0.15	0.05
Asian or Pacific Islander	2.0%	0.001	1.2%	0.003	17	-0.8%	0.039	0.06
Black/African American	9.6%	0.003	2.3%	0.004	33	-7.3%	< 0.001	0.33
Multiple Race	0.3%	0.001	0.1%	0.001	1	-0.2%	0.13	0.05
Other Race	0.4%	0.001	0.7%	0.002	10	0.3%	0.14	0.04
White	86.3%	0.004	94.9%	0.006	1368	8.5%	< 0.001	0.30
**Ethnicity†**								
Hispanic	9.3%	0.003	1.3%	0.003	19	-8.0%	< 0.001	0.39
Not Hispanic	90.7%	0.003	98.7%	0.003	1419			
**Employment**†***18–64 years old***								
Not Employed	44.8%	0.007	19.9%	0.013	178			
Employed	55.2%	0.007	80.1%	0.013	716	24.9%	< 0.001	0.54
***65 years or older***								
Not Employed	89.9%	0.006	80.4%	0.017	426			
Employed	10.1%	0.006	19.6%	0.017	104	9.6%	< 0.001	0.27
**Education†**								
Less than high school	20.9%	0.004	1.7%	0.003	24	-19.3%	< 0.001	0.69
High school	31.5%	0.005	20.4%	0.011	291	-11.1%	< 0.001	0.25
College, no degree	19.8%	0.004	21.5%	0.011	306	1.7%	0.13	0.04
Associate or technical school	10.9%	0.003	11.9%	0.009	170	1.0%	0.26	0.03
Bachelor’s	10.8%	0.003	28.6%	0.012	407	17.8%	< 0.001	0.46
Post-graduate or professional	6.1%	0.002	15.9%	0.010	227	9.9%	< 0.001	0.32
**Household Income†**								
$0 - $34,999	47.7%	0.006	29.5%	0.013	353	-18.2%	< 0.001	0.38
$35,000 - $74,999	31.5%	0.005	45.8%	0.014	548	14.3%	< 0.001	0.29
$75,000+	20.8%	0.005	24.7%	0.012	296	3.9%	0.002	0.09
**Body Mass Index†**								
Underweight	0.9%	0.001	0.6%	0.002	8	-0.3%	0.17	0.03
Healthy weight	28.0%	0.005	31.9%	0.012	461	3.9%	0.002	0.09
Overweight	35.8%	0.005	36.4%	0.013	526	0.6%	0.65	0.01
Obese	35.2%	0.005	31.1%	0.012	449	-4.2%	0.002	0.09
**Pain Duration†**								
1 year or less	9.9%	0.003	21.0%	0.011	296	11.2%	< 0.001	0.31
1 to 5 years	27.3%	0.004	32.8%	0.013	461	5.4%	< 0.001	0.12
5 to 10 years	22.0%	0.004	18.8%	0.010	265	-3.1%	0.007	0.08
Over 10 years	40.8%	0.005	27.4%	0.012	385	-13.4%	< 0.001	0.28
**Radiating Pain†**								
No	46.8%	0.005	60.0%	0.013	866			
Yes	53.2%	0.005	40.0%	0.013	578	-13.2%	< 0.001	0.27
**Diabetes†**								
No	87.0%	0.003	93.8%	0.006	1355			
Yes	13.0%	0.003	6.2%	0.006	89	-6.8%	< 0.001	0.23
**Smoking†**								
No	68.7%	0.005	90.3%	0.008	1304			
Yes	31.3%	0.005	9.7%	0.008	140	-21.6%	< 0.001	0.55
**Alcohol Use†**								
No	39.5%	0.005	41.5%	0.014	521			
Yes	60.5%	0.005	58.5%	0.014	735	-2.0%	0.18	0.04
SF-12/36 PCS*	40.5 (13.0)	0.196	43.0 (8.1)	0.216	1424	2.5	< 0.001	0.19
SF-12/36 MCS*	48.2 (11.5)	0.154	53.5 (9.1)	0.241	1424	5.3	< 0.001	0.46
QALYs*	0.69 (0.21)	0.003	0.76 (0.13)	0.004	1414	0.07	< 0.001	0.33

† Data from NHIS using 15,312 observations representative of 11,473,330 U.S. adults

*Data from MEPS using 12,679 observations representative of 15,604,791 U.S. adults

N = number; QALYs = Quality-adjusted life years; SD = standard deviation; SE = standard error

### Chiropractic use in U.S. population

Table 2 displays results of the multivariable logistic regression estimating the odds of chiropractic use for U.S. adults with chronic spine pain based on socio-demographic and clinical characteristics from the NHIS data. The odds of chiropractic use did not differ based on sex, ethnicity, body mass index, or the presence of diabetes. The odds of chiropractic use were significantly lower for adults from the southern U.S. (OR: 0.64 95%CI 0.54 to 0.75 relative to Northeast U.S.). Relative to young adults (18 to 30 years old), adults over the age of 50 have lower odds of chiropractic use (OR for 51 to 64 year olds: 0.70 95%CI 0.58 to 0.83). For race, Asian or Pacific Islander (OR: 0.68 95%CI 0.48 to 0.97) and Black adults (OR: 0.81 95%CI 0.68 to 0.96) have lower odds of chiropractic use compared to White adults, but no other racial differences were significant. For socio-economic status measures, the odds of chiropractic use significantly increased based on employment (compared to unemployed OR: 1.36 95%CI 1.21 to 1.52), higher education levels (compared to no high school degree; OR for some college, no degree: 1.41 95%CI 1.21 to 1.66), and higher household incomes (compared to household incomes <$35,000; OR for $75,000 + 1.19 95%CI 1.02 to 1.39). Adults with pain duration over a year had lower odds of chiropractic use relative to those with pain duration less than a year (OR for 5–10 years: 0.61 95%CI 0.50 to 0.73). The odds of chiropractic use also increased for those with radiating pain (OR: 1.18 95%CI 1.08 to 1.31), headaches (OR: 1.12 95%CI 1.01 to 1.25), alcohol users (OR: 1.16 95%CI 1.04 to 1.29), and non-smokers (OR for smokers: 0.69 95%CI 0.61 to 0.78).

**Table 2:** Odds of chiropractic use in U.S. Population with chronic spine pain using NHIS

	OR	95% CI		OR	95% CI
**Region of U.S.**			**Body Mass Index**		
Northeast	1.00	Reference	Underweight	0.76	(0.45 to 1.27)
Midwest	1.02	(0.86 to 1.21)	Healthy weight	1.00	Reference
South	0.64***	(0.54 to 0.75)	Overweight	0.96	(0.84 to 1.09)
West	1.06	(0.90 to 1.26)	Obese	0.89	(0.78 to 1.03)
**Age**			**Pain Duration**		
18 to 30	1.00	Reference	1 year or less	1.00	Reference
31 to 50	0.88	(0.75 to 1.03)	1 to 5 years	0.74***	(0.62 to 0.88)
51 to 64	0.70***	(0.58 to 0.83)	5 to 10 years	0.61***	(0.50 to 0.73)
65 to 84	0.69***	(0.56 to 0.85)	Over 10 years	0.63***	(0.54 to 0.75)
85 and above	0.45***	(0.29 to 0.70)	**Radiating Pain**		
**Sex**			No	1.00	Reference
Male	1.00	Reference	Yes	1.18***	(1.08 to 1.31)
Female	1.00	(0.90 to 1.11)	**Headache**		
**Race**			No	1.00	Reference
Alaskan Native, or American Indian	0.83	(0.55 to 1.26)	Yes	1.12*	(1.01 to 1.25)
Asian or Pacific Islander	0.68*	(0.48 to 0.97)	**Diabetes**		
Black/African American	0.81*	(0.68 to 0.96)	No	1.00	Reference
Multiple Race	0.43	(0.17 to 1.06)	Yes	0.89	(0.76 to 1.05)
Other Race	0.60	(0.33 to 1.10)	**Smoking**		
White	1.00	Reference	No	1.00	Reference
**Ethnicity**			Yes	0.69***	(0.61 to 0.78)
Hispanic	0.85	(0.72 to 1.02)	**Alcohol Use**		
Not Hispanic	1.00	Reference	No	1.00	Reference
**Employment**			Yes	1.16**	(1.04 to 1.29)
Not Employed	1.00	Reference	**Household Income**		
Employed	1.36***	(1.21 to 1.52)	$0 - $34,999	1.00	Reference
**Education**			$35,000 - $74,999	1.17**	(1.04 to 1.32)
Less than high school	1.00	Reference	$75,000+	1.19*	(1.02 to 1.39)
High school	1.20*	(1.02 to 1.40)			
College, no degree	1.41***	(1.21 to 1.66)			
Associate or technical school	1.39**	(1.13 to 1.70)			
Bachelor’s	1.26*	(1.02 to 1.55)			
Post-graduate or professional	1.30*	(1.02 to 1.65)			

*P≤0.05, **P≤0.01, ***P≤0.001

Data from NHIS using 15,312 observations representative of 11,473,330 U.S. adults

OR=odds ratio; CI=confidence interval

Table 3 displays results of the multivariable logistic regression estimating the odds of chiropractic use for U.S. adults with chronic spine pain based on socio-demographic and clinical characteristics from the MEPS data. The odds of chiropractic use were lower for adults 85 and older relative to younger adults (18 to 30 years old; OR 0.41 95%CI 0.22 to 0.75). For race and ethnicity, the odds of chiropractic use were lower for Asian or Pacific Islander (OR 0.59 95%CI 0.42 to 0.84), Black (OR: 0.27 95%CI 0.21 to 0.34), and Hispanic adults (OR: 0.37 95%CI 0.30 to 0.46). For overall physical and mental health, the odds of chiropractic use were increased for adults in the top three quartiles for physical health (OR for top quartile: 2.88 95%CI 2.10 to 3.94), the top two quartiles for mental health (OR to top quartile: 1.47 95%CI 1.11 to 1.94), and the middle two quartiles for quality-adjusted life years compared to those in the lowest quartile (OR for third quartile: 1.40 95%CI 1.03 to 1.90).

**Table 3:** Odds of chiropractic use in U.S. Population with chronic spine pain using MEPS

	OR	95% CI		OR	95% CI
**Age**			**SF-12/36 PCS**		
18 to 30	1.00	Reference	Quartile 1	1.00	Reference
31 to 50	1.04	(0.83 to 1.29)	Quartile 2	1.39**	(1.10 to 1.76)
51 to 64	1.03	(0.83 to 1.29)	Quartile 3	2.15***	(1.61 to 2.89)
65 to 84	0.87	(0.68 to 1.11)	Quartile 4	2.88***	(2.10 to 3.94)
85 and above	0.41**	(0.22 to 0.75)	**SF-12/36 MCS**		
**Sex**			Quartile 1	1.00	Reference
Male	1.00	Reference	Quartile 2	1.08	(0.87 to 1.35)
Female	1.22***	(1.09 to 1.36)	Quartile 3	1.36*	(1.07 to 1.74)
**Race**			Quartile 4	1.47**	(1.11 to 1.94)
Alaskan Native, or American Indian	0.57	(0.29 to 1.12)	**QALYs**		
Asian	0.59**	(0.42 to 0.84)	Quartile 1	1.00	Reference
Multiple Race	1.02	(0.66 to 1.57)	Quartile 2	1.34*	(1.05 to 1.71)
Other Race	0.58	(0.19 to 1.76)	Quartile 3	1.40*	(1.03 to 1.90)
White	1.00	Reference	Quartile 4	1.32	(0.93 to 1.88)
Black/African American	0.27***	(0.21 to 0.34)			
**Ethnicity**					
Hispanic	0.37***	(0.30 to 0.46)			
Not Hispanic	1.00	Reference			

*P≤0.05, **P≤0.01, ***P≤0.001

*Data from MEPS using 12,679 observations representative of 15,604,791 U.S. adults

OR=odds ratio; CI=confidence interval

### Trial participants relative to the US population visiting a chiropractor

Table 4 compares trial participants to the U.S. population with chronic spine pain and chiropractic use because all of the clinical trials included spinal manipulation, a treatment most commonly delivered by chiropractors in the U.S. Trial participants were still more likely to be older (21.7% more 65 to 84 year olds), female (3.4% more), and White (5.8% more), and less likely to be Black (5.3% less) or Hispanic (7.3% less) relative to the US population seeing a chiropractor. Socioeconomic status indicators such as employment (14.6% more in adults 18–64 years old), education (15.7% more with Bachelor degree), and household income (10.6% more with $35,000 to $74,999) were also higher among trial participants. For clinical characteristics, trial participants had shorter pain duration (7.1% more with duration less than one year), less radiating arm or leg pain (13.3% less), diabetes (3.4% less), smoking (16.6% less) or alcohol use (9.8% less). Overall physical health was lower in trial participants (1.9 lower SF-12/36 physical health score), mental health was higher (3.1 higher SF-12/36 mental health score), and there were no differences in quality-adjusted life years. Most differences were small in magnitude apart from small to medium differences in ethnicity and smoking and medium differences in age and education.

**Table 4:** U.S. Population with chronic spine pain who visited a chiropractor from NHIS & MEPS relative to trial participants

	U.S. Population who visited a chiropractor		Trial Population			Trial – U.S. Population		
	% or Mean (SD)	SE	% or Mean (SD)	SE	*N*	Difference	*P*-value	Effect Size
**Age†**								
18 to 30	13.7%	0.008	7.0%	0.007	101	-6.7%	< 0.001	0.22
31 to 50	44.7%	0.011	32.8%	0.012	473	-12.0%	< 0.001	0.24
51 to 64	26.3%	0.009	23.5%	0.011	339	-2.8%	0.057	0.06
65 to 84	14.2%	0.007	35.9%	0.013	518	21.7%	< 0.001	0.51
85 and above	1.1%	0.002	0.8%	0.002	12	0.3%	0.41	0.03
**Sex†**								
Male	44.1%	0.011	40.7%	0.013	588			
Female	55.9%	0.011	59.3%	0.013	856	3.4%	0.048	0.07
**Race†**								
Alaskan Native, or American Indian	1.2%	0.002	0.9%	0.002	13	-0.3%	0.46	0.03
Asian or Pacific Islander	1.8%	0.003	1.2%	0.003	17	-0.6%	0.13	0.05
Black/African American	7.6%	0.005	2.3%	0.004	33	-5.3%	< 0.001	0.25
Other Race	0.3%	0.001	0.7%	0.002	10	0.4%	0.026	0.06
Multiple Race	0.2%	0.001	0.1%	0.001	1	-0.1%	0.35	0.03
White	89.0%	0.006	94.9%	0.006	1368	5.8%	< 0.001	0.22
**Ethnicity†**								
Hispanic	8.7%	0.006	1.3%	0.003	19	-7.3%	< 0.001	0.37
Not Hispanic	91.3%	0.006	98.7%	0.003	1419			
**Employment**†***18–64 years old***								
Not Employed	34.6%	0.013	19.9%	0.013	178			
Employed	65.4%	0.013	80.1%	0.013	716	14.6%	< 0.001	0.33
***65 years or older***								
Not Employed	85.1%	0.018	80.4%	0.017	426			
Employed	14.9%	0.018	19.6%	0.017	104	4.7%	0.07	0.12
**Education†**								
Less than high school	14.2%	0.008	1.7%	0.003	24	-12.5%	< 0.001	0.51
High school	29.6%	0.010	20.4%	0.011	291	-9.2%	< 0.001	0.21
College, no degree	23.0%	0.009	21.5%	0.011	306	-1.5%	0.28	0.04
Associate or technical school	13.0%	0.008	11.9%	0.009	170	-1.1%	0.34	0.03
Bachelor’s	12.8%	0.008	28.6%	0.012	407	15.7%	< 0.001	0.40
Post-graduate or professional	7.3%	0.006	15.9%	0.010	227	8.6%	< 0.001	0.27
**Household Income†**								
$0 - $34,999	38.6%	0.011	29.5%	0.013	353	-9.1%	< 0.001	0.19
$35,000 - $74,999	35.2%	0.011	45.8%	0.014	548	10.6%	< 0.001	0.22
$75,000+	26.2%	0.011	24.7%	0.012	296	-1.5%	0.38	0.03
**Body Mass Index†**								
Underweight	0.7%	0.002	0.6%	0.002	8	-0.1%	0.63	0.01
Healthy weight	30.0%	0.010	31.9%	0.012	461	2.0%	0.22	0.04
Overweight	36.5%	0.011	36.4%	0.013	526	-0.1%	0.96	0.00
Obese	32.8%	0.010	31.1%	0.012	449	-1.7%	0.28	0.04
**Pain Duration†**								
1 year or less	13.9%	0.008	21.0%	0.011	296	7.1%	< 0.001	0.19
1 to 5 years	29.2%	0.010	32.8%	0.013	461	3.6%	0.028	0.08
5 to 10 years	19.9%	0.009	18.8%	0.010	265	-1.1%	0.42	0.03
Over 10 years	37.0%	0.010	27.4%	0.012	385	-9.6%	< 0.001	0.21
**Radiating Pain†**								
No	46.6%	0.011	60.0%	0.013	866			
Yes	53.4%	0.011	40.0%	0.013	578	-13.3%	< 0.001	0.27
**Diabetes†**								
No	90.4%	0.006	93.8%	0.006	1355			
Yes	9.6%	0.006	6.2%	0.006	89	-3.4%	< 0.001	0.13
**Smoking†**								
No	73.7%	0.010	90.3%	0.008	1304			
Yes	26.3%	0.010	9.7%	0.008	140	-16.6%	< 0.001	0.44
**Alcohol Use†**								
No	31.7%	0.010	41.5%	0.014	521			
Yes	68.3%	0.010	58.5%	0.014	735	-9.8%	< 0.001	0.20
SF-12/36 PCS*	44.9 (11.7)	0.323	43.0 (8.1)	0.216	1424	-1.9	< 0.001	0.16
SF-12/36 MCS*	50.4 (10.0)	0.284	53.5 (9.1)	0.241	1424	3.1	< 0.001	0.31
QALYs*	0.762 (0.17)	0.005	0.760 (0.13)	0.004	1414	0.002	0.70	0.01

† Data from NHIS using 3006 observations representative of 2,315,852 U.S. adults

*Data from MEPS using 2870 observations representative of 4,194,660 U.S. adults

MCS = mental component score; N = number; PCS = physical component score; QALYs = Quality-adjusted life years; SD = standard deviation; SE = standard error

## Discussion

### Summary of findings

We identified important differences between clinical trial participants and the general U.S. population with chronic spine pain. The clinical trials had an over-representation of White, non-Hispanic, employed participants with higher household incomes relative to the general US population with chronic spinal pain. Trial participants also had shorter pain duration, less radiating pain, fewer co-morbid conditions, worse physical health, and better mental health. All of the clinical trials included spinal manipulation, a common modality used by chiropractors in the U.S. The odds of chiropractic use in the U.S. are lower for older adults, Black or Asian/Pacific Islander adults, the unemployed, those with less education, lower household income, longer pain duration, and a history of smoking. The odds of chiropractic use also decreased for those with lower physical or mental health. Despite these differences between people who receive and do not receive chiropractic care, there were similar important differences compared to clinical trial participants when limiting the U.S. population to adults with chronic spinal pain who visited a chiropractor in the past year. Most of the differences identified had small effect sizes, with small to medium or medium effect sizes for age, ethnicity, employment, income, education, smoking status, and mental health.

### Strengths and limitations

This study has important strengths and weaknesses. First, the use of individual participant data from multiple clinical trials measuring similar demographic and health characteristics that could be combined and compared to national survey measures is an important strength. Existing studies pooling individual participant data from spine pain trials have noted substantial heterogeneity between studies in demographic and health characteristics collected with sex and age being the only two characteristics consistently collected and reported [24]. Another strength of this study was the use of nationally representative surveys, where demographic and health characteristics of adults with chronic spine pain were available. Besides these strengths, this project also has important limitations. The clinical trial data used for this project was limited to a readily available sample of trials conducted by one research group with most participants recruited from a single geographic location. This limitation can potentially impact generalizability of this study’s findings if spine pain trials conducted by other groups included a more diverse sample of participants who are more representative of the population of interest. While we were able to identify individuals with chronic spine pain in all three data sources, the chronic pain indicators and definitions were different (RCTs: 3 month or longer duration of neck or back pain/problem; NHIS: 3 month or longer duration of neck or back pain/problem causing functional limitations; MEPS: two separate healthcare visits for neck or back problem at least 5 months apart over 2.5 years). Also, health characteristics were self-reported and are subject to potential recall bias. Health characteristics were, however, self-reported in the clinical trials and national health surveys, so any potential misclassifications would not be expected to have a differential impact. Finally, there were slight differences in time periods between the data sources used (NHIS: 2001–2010; MEPS: 2002–2010; Trials: Majority 2000–2010, one trial from 1994 to 1997 and one trial from 2010 to 2013).

### Comparisons to other research

This is the first study comparing demographic and clinical characteristics of participants in clinical trials for spinal pain to the U.S. population. However, several individual patient data meta-analyses (IPDMA) of spinal pain treatments provide a summary of the typical population in clinical trials for spine pain, including two trials included in this project [10, 11] and other trials within and outside of the U.S [24–26]. The broader set of international clinical trials for spine pain have demographic characteristics similar to those in the eight trials used for this analysis in terms of sex (∼ 55% female), race and ethnicity (90% White, non-Hispanic), employment (51–75% employed), and education (68% low/middle education). Clinical characteristics such as duration of spine pain (20% <1 year) and presence of leg pain (59%) were also similar. One notable difference compared to existing IPDMA trials is that participants in the trials used for this project reported better overall mental health as indicated by higher scores on the SF-36 mental component summary (53.5 in the 8 trials vs. 45 in existing IPDMAs). It is unclear why this difference exists as the inclusion/exclusion criteria for the eight trials included in the study are similar to the trials included in the IPDMAs (e.g., exclude individuals with severe comorbid conditions, substance abuse).

### Implications for clinical practice

The important differences between trial participants and the U.S. population with chronic spinal pain raises questions regarding how findings from the trials may generalize to inform clinical practice. So long as typical patients seen by a provider reflect the trial populations (e.g., predominantly white, educated, employed, with few comorbidities) the clinical trials’ findings will readily translate. For populations under-represented in clinical trials, the findings may still be relevant, so long as the treatment effects are not modified by characteristics of the population. Research investigating effect modification of spinal pain treatments is an emerging field as clinical trials are not typically powered for such analyses and IPDMA have limitations due to inconsistent availability and measurement of potential modifying factors.

Three recent IPDMAs assessed potential treatment effect modifiers of non-invasive and non-pharmacological treatments such as spinal manipulation and exercise for low back pain [24–26]. These studies found that younger individuals with worse disability, physical health status, less psychological distress, and shorter pain duration were more likely to benefit from passive physical treatments including spinal manipulation. The absence of heavy physical demands at work, lower body mass index, and medication use for low back pain (LBP) were potential effect modifiers favoring exercise interventions. Education and employment did not moderate the effect of spinal manipulation or exercise interventions. In the current study, we found differences in some of these effect modifying characteristics. Relative to the U.S. population with chronic spine pain, the clinical trial populations had an over-representation of individuals with shorter pain duration (less than 1 year), better physical health, and better mental health. Effect modification studies have shown that individuals with shorter pain duration and better mental health show a larger benefit with spinal manipulation and those with better physical health have less benefit relative to other treatments.

### Implications for research

Although greater attention has been devoted to increasing the diversity and representativeness of clinical trial participants, many trials still fall short. Inclusion of racial and ethnic minority groups in NIH-funded clinical trials has increased over the past 25 years (from 2.8 to 11.1%), but they are still widely underrepresented [27]. Representation of Black and Hispanic adults in clinical trials for pain treatments are lower than census estimates by a factor of 2 to 3.5 [28, 29]. This under-representation is especially troubling given known disparities in health outcomes. NIH-designated health disparity populations in the U.S. include racial and ethnic minority groups, sexual and gender minorities, socioeconomically disadvantaged populations, and underserved rural populations [30]. Among individuals with chronic pain, Black Americans and individuals in the lowest wealth quartile report more pain related disability [31]. Further, high impact chronic pain, pain that limits life activities or work on most days, is twice as prevalent among those with low income [31].

Several factors contribute to the low participation of health disparity populations in clinical research. These include low research literacy, lack of culturally relevant information in the consent process, and distrust of researchers, including fears that participation will worsen their health status, expose them to unnecessary risks, lead to a loss of privacy or confidentiality, or result in stigmatization [32, 33]. Further, the burden of clinical research participation is often a significant barrier as transportation, financial, and time demands are greater than routine clinical care [32]. Also, biases among those who recruit and screen for clinical trials is a barrier, as individuals from health disparity populations can be viewed as less than ideal candidates due to potential concerns about attending study visits and complying with treatment and data collection protocols [34].

To enact change, spine pain researchers need to embrace key community-based approaches that have shown promise for increasing participation of health disparity populations [35]. These approaches are becoming common in many health research fields but are used infrequently in pain research [36–38]. Successful strategies are often multilevel, engage the community throughout the research process, and address concerns at the participant, provider, and community level, including issues of trust [36]. Strategies that have been used successfully by others include engaging key community members in trial protocols and implementation early and often within the project’s life cycle, use of patient navigators to reduce the complexity of participation, pilot testing of recruitment approaches, placing enrollment and treatment sites in underserved communities to reduce travel burden, and use of flexible appointment times to reduce work or childcare barriers to participation [36, 39].

## Conclusions

This project assessed the generalizability of randomized trial populations participating in research assessing spinal manipulation for chronic spinal pain. Compared to the U.S. population with spine pain, the clinical trials had an under-representation of individuals from health disparity populations with lower percentages of racial and ethnic minorities and people who were less educated or unemployed. While the odds of chiropractic use in the U.S. are lower for individuals from health disparity populations, the trial populations also under-represented these populations relative to U.S. adults with chronic spine pain who visit a chiropractor.

### Electronic supplementary material

Below is the link to the electronic supplementary material.

Supplementary Material 1

## Data Availability

Study data are available from the corresponding author by reasonable request.

## Abbreviations

MCS SF-12: Mental Component Summary Score
MEPS: Medical Expenditure Panel Survey
NHIS: National Health Interview Survey
PCS SF-12: Physical Component Summary Score
QALYs: Quality-adjusted Life Years
RCTs: Randomized clinical trials
U.S.: United States
