# Supplementary material for: How well do participants in clinical trials represent the U.S. population with chronic neck or back pain?
Source: BMC Musculoskelet Disord. 2024 May 27;25:414. doi: 10.1186/s12891-024-07524-9 (PMC11129496; doi:10.1186/s12891-024-07524-9)
Supplement: Supplementary file 1 — Supplementary Material 1 [file 12891_2024_7524_MOESM1_ESM.docx]

**Appendix Materials**

Appendix Table 1. NHIS and MEPS variables

| Source | Variable Name | Description |
| --- | --- | --- |
| IPUMS NHIS | AGE | Age in years since their last birthday |
|  | SEX | Indicates whether the person was male or female |
|  | RACEA | Racial background using the pre-1997 Office of Management and Budget’s (OMB’s) Statistical Policy Directive No. 15. |
|  | HISPETH | Hispanic/Spanish/Latino origin or ancestry based on the origin of a parent, grandparent, or some far-removed ancestor. |
|  | EDUC | Highest level of schooling completed |
|  | EMPSTAT | Employment status in the last 1-2 weeks |
|  | INCFAM97ON2 | Total household income using categories introduced in 2007 |
|  | BMICALC | Calculated body mass index |
|  | SAWCHIR | Identifies individuals who had seen or talked to a chiropractor during the past 12 months |
|  | DIABETICEV | Identifies individuals who had ever been diagnosed with “diabetes or sugar diabetes” by a doctor or other health professional |
|  | LEGPAIN3MO | Identifies individuals who had low back pain that “spread down either leg to areas below the knees” during the past three months |
|  | ALCSTAT1 | Identifies individuals who had at least 12 drinks of any type of alcoholic beverage in their lifetime |
|  | SMOKESTATUS2 | Identifies current smoking status as every day, some days, unknown frequency, former smoker, and never smoked. |
|  | FLBACKC | Indicates whether adults who have at least a little difficulty with one or more functional activities due to a back or neck problem have a chronic back or neck problem |
|  | FLBACKNO/  FLBACKTP | Duration of back or neck problem causing at least a little difficulty with one or more functional activities |
| MEPS | CCC0DEX | Aggregation of ICD-9-CM condition codes into clinically meaningful categories that group similar conditions |
|  | PCS42 | Physical Component Summary of the SF-12 Version 2 |
|  | MCS42 | Mental Component Summary of the SF-12 Version 2 |
|  | CHIROVISIT | Number of office-based visits to a chiropractor |
|  | ADDAYA42 | Limitations in moderate activities during a typical day (used to construct SF6D) |
|  | ADPWLM42 | Limitations in kind of work and other activities due to physical health in past four weeks (used to construct SF6D) |
|  | ADMALS42 | Accomplished less than would like due to mental problems in the past four weeks (used to construct SF6D) |
|  | ADSOCA42 | Physical health or emotional problems interfered with social activities during the past four weeks (used to construct SF6D) |
|  | ADPAIN42 | Pain interfered with normal work outside the home and housework during past four weeks (used to construct SF6D) |
|  | ADDOWN42 | Felt downhearted and depressed during past four weeks (used to construct SF6D) |
|  | ADNRGY42 | Had a lot of energy during the past four weeks (used to construct SF6D) |
